# Rethinking Drug Resistance in Acute Promyelocytic Leukemia: The Regulated Cell Death Network as an Integrative Framework

**DOI:** 10.3390/biom16091375

**Published:** 2026-09-21

**Authors:** Shiyu Lin, Biaohan Qin, Zhexi Ye, Jiaqi Qing, Hui Zhen, Sennan Yang, Xin Jiang, Yiwen Fan, Lemei Zhu, Juan Liu

**Affiliations:** 1 Hunan Key Laboratory of the Research and Development of Novel Pharmaceutical Preparations, Changsha Medical University, Changsha 410219, China; 2 Hunan Provincial University Key Laboratory of the Fundamental and Clinical Research on Neurodegenerative Diseases, Changsha Medical University, Changsha 410219, China; 3 School of Traditional Chinese Medicine, Changsha Medical University, Changsha 410219, China; 4 School of First Clinical, Changsha Medical University, Changsha 410219, China; 5 School of Public Health, Changsha Medical University, Changsha 410219, China; 6 School of Dental Medicine, Changsha Medical University, Changsha 410219, China

**Keywords:** APL, ATRA/ATO resistance, RCDN, network remodeling, mitochondrial priming, redox signaling, Tan IIA

## Abstract

Drug resistance in acute promyelocytic leukemia (APL) remains clinically challenging despite the success of all-trans retinoic acid (ATRA) and arsenic trioxide (ATO) therapy. Existing explanations, including promyelocytic leukemia protein–retinoic acid receptor alpha (PML–RARα) mutations and alterations in individual death pathways, do not fully account for the heterogeneity of resistant phenotypes. This review proposes regulated cell death network (RCDN) remodeling as a testable framework for APL resistance. RCDN remodeling is defined as coordinated changes in network coupling, stress routing, and cellular death thresholds. We hypothesize that resistance emerges from coordinated adaptation across redox, survival signaling, and mitochondrial gating, rather than from defects in any single pathway. This predicts coupled shifts across multiple death modules and non-additive responses to combinatorial perturbations, distinguishing network-level remodeling from pathway-specific resistance. We further define experimental criteria for distinguishing network-level remodeling from pathway-specific resistance and discuss Tanshinone IIA (Tan IIA) as a preclinical multi-node perturbation probe.

## 1. Introduction

APL is a distinct subtype of acute myeloid leukemia (AML) characterized by the t(15;17) (q24;q21) translocation and the resulting PML-RARα fusion oncoprotein. PML-RARα represses retinoic acid-dependent transcription, blocks the terminal differentiation of promyelocytes, and disrupts the organization and tumor-suppressive functions of PML nuclear bodies (PML-NBs) [1,2]. ATRA and ATO have transformed APL into one of the most curable acute leukemias by targeting complementary components of PML-RARα [1,3,4]. ATRA acts through the RARα moiety to relieve transcriptional repression and restore differentiation, whereas ATO targets the PML moiety, promoting PML-RARαdegradation, PML-NB reorganization, oxidative stress, and apoptosis [3,4]. Treatment is risk-adapted: patients with non-high-risk disease (white blood cell count ≤ 10 × 10^9^/L) generally receive ATRA plus ATO as a chemotherapy-free regimen, whereas those with high-risk disease (>10 × 10^9^/L) usually receive additional cytoreductive treatment with an anthracycline or gemtuzumab ozogamicin. ATRA plus anthracycline-based chemotherapy is also an established option [1,5]. Thus, ATRA and ATO are definitive antileukemic agents rather than drugs used solely for sensitization.

In this review, resistance denotes reduced cellular responsiveness to ATRA- and ATO-induced differentiation or death signals. Clinically, it may manifest as primary refractory disease—failure to achieve hematological remission after induction or molecular remission after consolidation—or as relapse after an initially documented remission. Modern ATRA/ATO-based regimens achieve complete remission in approximately 95–100% of patients who survive induction; primary refractoriness is therefore uncommon, occurring in approximately 0–5% of evaluable patients [1,5,6]. Relapse occurs in approximately 5–10% of patients overall, depending on disease risk and treatment regimen [5]. ”Treatment failure” refers here to either refractory disease or relapse. Early death from hemorrhage, coagulopathy, differentiation syndrome, or infection is considered separately and is not classified as biological evidence [1,6]. This review focuses on adaptations that permit survival during ATRA/ATO exposure; evidence involving other drugs is included only when it clarifies relevant mechanisms and is not treated as direct evidence of clinical resistance to ATRA/ATO. Resistance to APL therapy has been associated with alterations of PML-RARα, impaired differentiation, dysregulated survival signaling, metabolic adaptation, and altered susceptibility to regulated cell death (RCD) [7,8,9,10,11,12,13]. PML-RARα mutations have been associated with reduced responsiveness to ATO [14], but stable genetic lesions do not explain all cases. Non-genetic and potentially reversible states, including metabolic reprogramming and drug-tolerant phenotypes, may also contribute to resistance [15,16,17,18,19,20,21,22]. Although mechanistically diverse, these alterations converge on whether treatment-induced stress crosses the threshold for irreversible cell death.

RCD modalities, including apoptosis, ferroptosis, and necroptosis, have distinct execution mechanisms but share regulatory inputs involving redox homeostasis, metabolism, survival signaling, and mitochondrial function [23,24,25,26,27,28]. ATO illustrates this coupling in APL: its interaction with PML-RARα promotes PML modification and PML-NB reorganization, while PML-associated processes participate in oxidative-stress sensing and p53 activation [4,29,30,31,32]. These findings provide a rationale for examining resistance through interconnected regulatory processes rather than isolated pathways.

Against this background, RCDN remodeling refers to persistent changes in network coupling, stress routing, and cellular death thresholds that may alter the balance between adaptation and irreversible cell death. It extends the concept of RCD plasticity, which describes the capacity of cells to alter death responses under changing conditions [26,33,34]. The framework primarily addresses cell death regulation during ATRA/ATO exposure, particularly ATO-associated stress processing; isolated defects in ATRA-induced differentiation are considered only when they alter death susceptibility. RCDN remodeling is presented as a hypothesis-generating framework rather than an established mechanism, biomarker system, or therapeutic model in APL. Its validation will require temporal and perturbational evidence showing that coordinated network changes precede or sustain resistance and provide greater explanatory value than individual pathway alterations.

**Figure 1:**
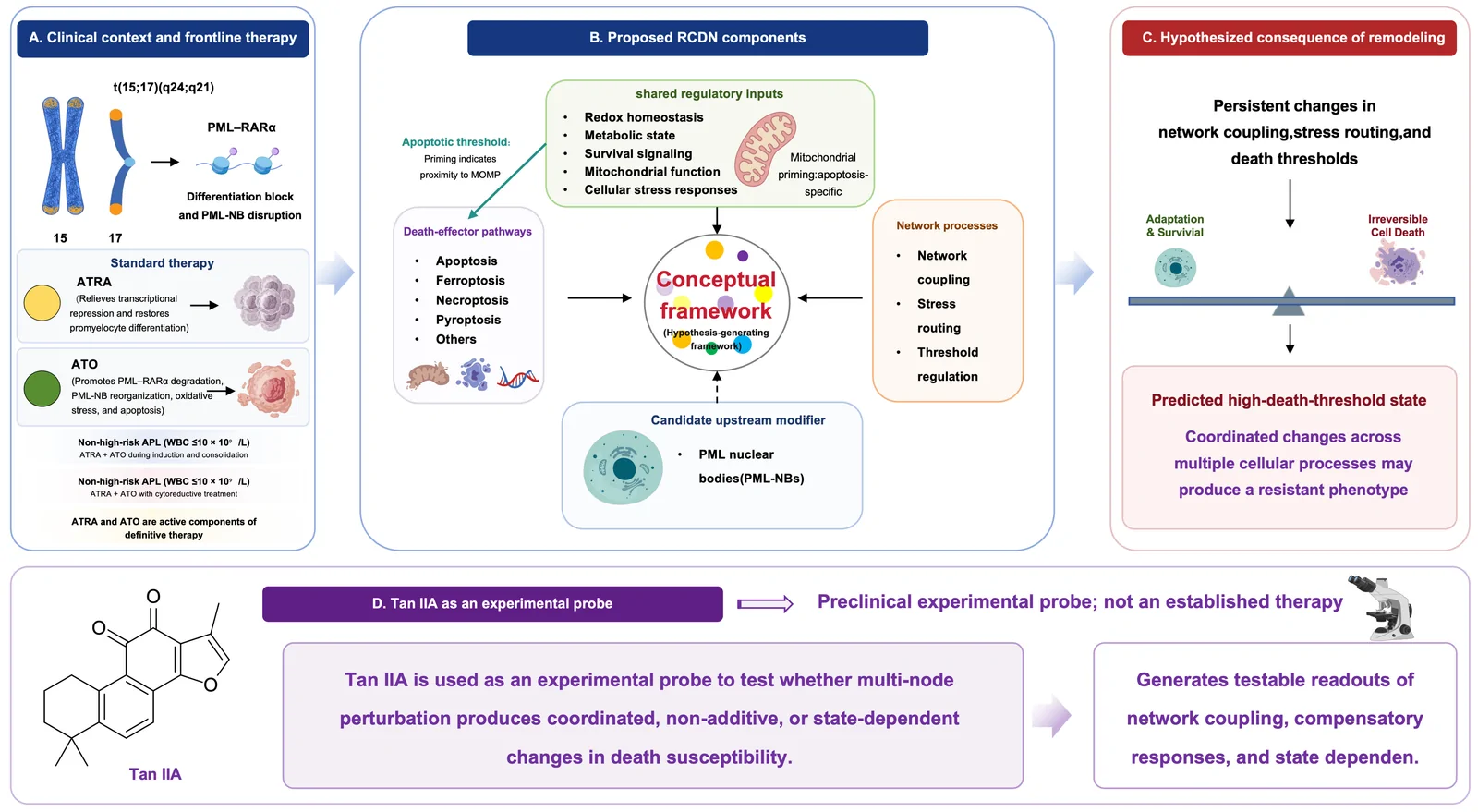
Hypothesized RCDN remodeling in APL and its experimental interrogation using Tan IIA. (**A**) PML–RARα-associated abnormalities, mechanisms of ATRA/ATO, and risk-adapted frontline therapy. (**B**) Proposed RCDN components, with mitochondrial priming/MOMP specifically defining the apoptotic threshold. (**C**) Hypothesized transition toward a high-death-threshold resistant state. (**D**) Tan IIA as a preclinical multi-node perturbation probe for evaluating network coupling, compensatory responses, and state dependence.

Tanshinone IIA (Tan IIA) is introduced as a multi-node perturbation probe (Figure 1). Its reported effects on redox regulation, phosphoinositide 3-kinase/protein kinase B/mechanistic target of rapamycin (PI3K/Akt/mTOR) signaling pathway, mitochondrial function, autophagy, and apoptosis allow testing of coordinated, non-additive, or state-dependent changes in death susceptibility. Responses during exposure and after washout may also help distinguish reversible plasticity from persistent remodeling. Tan IIA is not proposed as an established treatment for resistant APL; its supporting evidence and pharmacological limitations are discussed in Section 3.5.

## 2. Materials and Methods

Literature was identified through searches of PubMed/MEDLINE, Web of Science, and Scopus, supplemented by manual screening of reference lists from key publications. Searches covered publications from database inception through 2025 and included terms related to APL, ATRA/ATO resistance, regulated cell death, reactive oxygen species (ROS) and redox signaling, mitochondrial function, PI3K/Akt signaling, PML nuclear bodies, and Tan IIA. Priority was given to primary studies directly addressing APL biology, treatment response, or resistance, with mechanistic studies from non-APL AML and other cancer models considered when direct evidence in APL was limited. Evidence was organized according to disease relevance and mechanistic strength, with findings derived directly from APL models distinguished from evidence extrapolated from AML or other cancer systems. As a narrative review, the literature selection emphasized conceptual and mechanistic relevance rather than a systematic assessment of all available studies.

## 3. Results

### 3.1. Defining the RCDN in APL Resistance

Throughout this section, evidence is evaluated hierarchically. Non-APL evidence supports plausibility and hypothesis generation, but it does not demonstrate that the same network operates in APL. Such evidence is contextual rather than direct. Direct evidence from APL models or patient samples is prioritized when discussing APL resistance, while findings from broader AML studies serve to provide mechanistic context, and data from other cancer systems are used only to support conserved cellular mechanisms or generate testable hypotheses. Evidence derived from AML or non-hematological cancer models is therefore not interpreted as direct evidence of RCDN remodeling in APL.

#### 3.1.1. Definition and Architecture of the RCDN

Here, RCDN denotes a functional organization of interconnected regulatory and death-effector modules that influence cellular susceptibility to irreversible death. It integrates regulated cell death modalities with upstream processes that control their engagement, including redox regulation, survival signaling, mitochondrial function, metabolism, and stress responses. Its defining feature is the functional coupling among these modules and the resulting effects on stress processing and death thresholds.

Although the RCDN framework is conceptually applicable to other malignancies, this Perspective focuses specifically on its potential relevance to APL drug resistance. Evidence from AML and other cancer systems is therefore used to provide mechanistic context rather than to establish RCDN remodeling in APL.

Apoptosis, ferroptosis, necroptosis, pyroptosis, and other regulated cell death (RCD) modalities are governed by distinct molecular execution mechanisms [24,25,26,33,35]. Their activation, however, is influenced by shared regulatory processes, including redox balance, mitochondrial function, metabolism, calcium signaling, stress responses, and survival signaling [28,36,37,38]. The RCDN is therefore defined as a functional network comprising multiple RCD modalities and the interconnected cellular processes that regulate their activation, coupling, and execution.

Conceptually, the RCDN consists of an execution layer and a regulatory layer. The execution layer includes apoptosis, ferroptosis, necroptosis, pyroptosis, and other established RCD modalities, whereas the regulatory layer comprises redox control, mitochondrial function, metabolism, calcium and stress signaling, and survival pathways [26,28,35,36,38,39,40,41,42,43]. Autophagy is considered here primarily as a stress-adaptation and network-modulating process, rather than as a canonical RCD modality equivalent to apoptosis or ferroptosis [44]. The interaction between these layers determines how cellular stress is processed and whether it reaches an irreversible death threshold.

The RCDN state is determined by the functional relationships among regulatory and execution modules and by their effects on cellular responses to defined stress inputs. The same therapeutic stress may therefore produce different outcomes depending on cellular state. In APL, ATO-induced oxidative stress is directly relevant to therapeutic responses, whereas broader studies across AML and other cancer systems indicate that ROS can also engage adaptive redox responses and modify death susceptibility [13,21,29,39,45,46,47]. These broader findings provide mechanistic context for the RCDN framework but should not be interpreted as direct evidence that the same network configuration occurs in APL.

For APL resistance, we focus on three functional dimensions: redox adaptation, survival signaling reinforcement, and mitochondrial gating. These dimensions describe how cellular stress is buffered, transmitted, and ultimately coupled to death execution. These are heuristic vantage points, not an alternative topology: redox adaptation reflects oxidative buffering [21,45,46,48]; survival signaling reflects pro-survival output intensity (e.g., PI3K/Akt) [11,12]; mitochondrial gating reflects the threshold for irreversible apoptotic commitment, marked by mitochondrial outer membrane permeabilization (MOMP) [27]. Because these dimensions overlap and interact bidirectionally, the “three-layer” language in the Highlights and Abstract refers to this functional tripartition, not to a revised network architecture.

RCD crosstalk describes interactions between death pathways, whereas RCDN focuses on how these interactions operate within a broader regulatory state that determines stress processing and death susceptibility [35,48].

#### 3.1.2. Conceptual Boundaries of the RCDN Framework

The RCDN differs from related concepts in its focus on functional coupling among regulatory modules and on how these interactions shape cellular death thresholds. The distinctions from RCD crosstalk, mitochondrial priming, drug-tolerant persistence, non-genetic resistance, PANoptosis, and static RCD network maps are summarized in Table 1.

Mitochondrial priming describes how close a cell is to undergoing MOMP, an event that usually marks commitment to intrinsic apoptosis. It is governed largely by the balance between pro-apoptotic and anti-apoptotic B-cell lymphoma 2 (BCL-2) family proteins and can be assessed using BCL-2 homology 3 (BH3) profiling [27]. Highly primed cells require less additional stress to undergo MOMP than poorly primed cells. Within the RCDN framework, mitochondrial priming is an apoptosis-specific readout rather than a general measure of susceptibility to non-apoptotic death programs.

#### 3.1.3. What Does RCDN Remodeling Mean?

Evidence for RCDN remodeling rests not on molecular changes per se, but on altered functional coupling among modules or shifted cellular responses to stress inputs [26,34]. RCDN remodeling can involve changes in the coupling among redox regulation, survival signaling, mitochondrial function, metabolism, and death-effector pathways. These changes may alter how a given therapeutic stress is processed and routed toward adaptive survival, recovery, or irreversible death [38,39,40,41,42,43,47]. Accordingly, stress routing refers to the functional disposition of a given stress signal across the network, whereas death threshold refers to the magnitude or combination of stress required to commit a cell to irreversible death. A resistant state may therefore arise without complete loss of any individual death pathway if network coupling is altered such that therapeutic stress is preferentially buffered or redirected away from death execution.

RCDN remodeling can be distinguished from simple pathway switching. A shift in the predominant death modality may occur as a consequence of network remodeling, but switching from one RCD pathway to another is neither necessary nor sufficient to establish remodeling. Likewise, transient pathway activation or reversible changes in drug sensitivity do not by themselves demonstrate remodeling. Evidence for RCDN remodeling should instead include reproducible changes in functional coupling, stress routing, or death thresholds that alter cellular susceptibility to defined perturbations. Such functional criteria are consistent with the distinction between transient changes in cell death responses and more persistent alterations in cellular death-state regulation [34].

This concept also differs from RCD plasticity. Plasticity denotes the capacity of a cell to alter its death response, whereas remodeling refers to a persistent or functionally meaningful reorganization of the interactions governing that response [33,34]. Thus, remodeling represents a change in the functional organization of the network, rather than simply the presence of molecular alterations or the transient activation of individual death pathways.

The RCDN framework addresses cell death evasion in APL resistance rather than differentiation blockade alone. Although pure ATRA resistance lies outside its scope, ATRA may indirectly shape the context in which ATO-induced death signals operate. The framework therefore centers on death-threshold regulation under ATRA/ATO combination therapy.

#### 3.1.4. RCDN Remodeling as a Model of APL Drug Resistance

A central hypothesis addressed here is that APL drug sensitivity and resistance represent different functional states of the same RCDN, rather than the simple presence or absence of individual death pathways. In treatment-sensitive cells, ATRA/ATO-induced stress may exceed cellular buffering capacity and propagate toward mitochondrial commitment and downstream death execution. In resistant cells, enhanced redox buffering, survival signaling, mitochondrial protection, or metabolic adaptation may attenuate or redirect these signals, such that comparable therapeutic stress fails to reach the threshold for irreversible death [21,45,46,49,50,51,52,53,54,55,56,57].

Accordingly, the predicted difference between sensitive and resistant APL cells is not necessarily the magnitude of the initial stress input, but how that input is processed and routed through the RCDN. Resistant cells would therefore be expected to exhibit altered death thresholds, adaptive responses, or functional coupling among redox, survival, mitochondrial, and death-effector modules. Importantly, these changes need not occur uniformly across all RCD modalities. A resistant state may retain apoptotic competence while showing altered susceptibility to ferroptosis or other non-apoptotic death programs, reflecting redistribution of stress toward alternative cellular responses rather than global loss of death competence [28,33,38].

Conversely, sufficiently strong or appropriately combined perturbation of complementary network nodes would be expected to restore death susceptibility if resistance reflects network-level buffering rather than irreversible loss of death competence. This prediction is consistent with the broader observation that metabolic and non-genetic adaptations can contribute to ATO resistance and treatment persistence, while remaining distinct from established genetic resistance mechanisms [21,22].

Redox adaptation, PI3K/Akt signaling, mitochondrial protection, and alternative RCD pathways may each contribute to resistance, but their functional coupling may be required to explain the resistant state (Table 2).

#### 3.1.5. Predictions That Distinguish RCDN Remodeling from Simpler Models

The RCDN framework leads to several predictions that can be tested experimentally. First, resistant APL cells would be expected to exhibit coordinated but non-uniform changes in susceptibility across multiple RCD modalities, rather than a uniform suppression of all death pathways. Second, at matched intracellular stress inputs, resistant cells would be expected to show altered stress processing, such as enhanced adaptive responses or delayed death execution, rather than simply reduced stress generation [21,39,47]. Third, perturbation of one regulatory node would be predicted to alter the functional response of another if these modules are genuinely coupled. Fourth, combined perturbations would be predicted to produce non-additive or sequence-dependent effects if network interactions contribute to the resistant state. Finally, transient perturbation followed by washout would be expected to distinguish reversible pathway effects from more persistent network-state stabilization.

Conversely, the RCDN model would be weakened if resistance were fully explained by a single molecular lesion, if redox, survival, mitochondrial, and death-effector pathways behaved independently and additively, or if functional measures of death susceptibility provided no information beyond established molecular markers. These criteria distinguish RCDN remodeling from a descriptive aggregation of resistance-associated pathways and provide a basis for its experimental falsification.

### 3.2. From Individual Pathways to RCDN Remodeling

#### 3.2.1. Redox Adaptation: An Established Feature with Unresolved Directionality

ATO-induced oxidative stress contributes to PML–RARα degradation and leukemic cell elimination [3,4,13,29]. In treatment-sensitive cells, excessive ROS can amplify mitochondrial damage, lipid peroxidation, DNA damage, and downstream death signaling. Conversely, metabolic adaptation and altered redox homeostasis have been implicated in ATO-resistant APL, with increased antioxidant capacity and enhanced buffering of oxidative stress reported in resistant states [22,45,46]. However, evidence directly linking these redox adaptations to coordinated remodeling of multiple RCD modalities in APL remains limited (Table 2).

The relationship between ROS and cell fate is intrinsically context-dependent. The consequences of oxidative stress vary according to its magnitude, duration, subcellular localization, metabolic context, and the capacity of cells to engage compensatory responses [39,47]. Thus, the relevant RCDN question is not simply whether ROS increases, but how oxidative signals are processed within a given cellular state. Oxidative stress may propagate toward mitochondrial dysfunction, lipid damage, and irreversible death, or may instead be buffered and coupled to adaptive programs involving NRF2-dependent antioxidant responses, metabolic remodeling, and PI3K/Akt-associated survival signaling. The resulting phenotype may therefore depend on the balance between oxidative injury and compensatory capacity rather than on ROS abundance alone. This provides a mechanistic rationale for viewing redox adaptation as a network component rather than an isolated resistance pathway.

#### 3.2.2. Survival Signaling Reinforcement: A Context-Dependent Regulatory Node

PI3K/Akt and MAPK signaling have been implicated in APL resistance, while studies in broader AML and other cancer systems provide additional evidence that these pathways can reinforce survival, metabolic adaptation, and stress tolerance [11,58,59,60]. The latter evidence is best regarded as mechanistic support rather than direct evidence of RCDN remodeling in APL. Importantly, Akt activation is not necessarily a static molecular marker of resistance. Its functional consequences depend on signal intensity and duration, feedback through downstream effectors, basal redox state, and cellular metabolic context [58,59,60].

Within the RCDN framework, Akt can be conceptualized as a regulatory node that couples stress adaptation to death susceptibility. Its contribution is better evaluated functionally than inferred from pathway activation alone. For example, transient sensitization following Akt inhibition would be more consistent with a reversible pathway effect, whereas a sustained alteration in death susceptibility after prolonged perturbation would provide stronger evidence for feedback stabilization. Even in the latter case, however, such findings would support rather than independently establish RCDN remodeling.

#### 3.2.3. Mitochondrial Regulation and Death-Effector Engagement

Mitochondrial priming describes how close mitochondria are to MOMP, whereas MOMP is the membrane-permeabilization event itself. When pro-apoptotic signaling exceeds the buffering capacity of anti-apoptotic BCL-2 family proteins, MOMP releases cytochrome c and other apoptogenic factors and commits the cell to intrinsic apoptosis [27,35,56,61,62,63,64]. Alterations in BCL-2 family regulation, mitochondrial quality control, and metabolic state can therefore alter priming and change the amount of additional stress required to trigger MOMP [43,61,62,63,64,65,66,67,68]. We refer to this shift as mitochondrial gate repositioning.

Mitochondrial priming is directly associated with intrinsic apoptosis and should not be regarded as a universal threshold for all RCD modalities. Ferroptosis, for example, is primarily driven by iron-dependent lipid peroxidation and failure of antioxidant defenses and can proceed without canonical MOMP-mediated apoptosis [28,38]. Nevertheless, mitochondrial redox activity, metabolism, and quality-control mechanisms can influence the cellular conditions that determine susceptibility to ferroptosis and other death programs [39,42,43]. Within the RCDN, mitochondria may therefore function as an apoptosis-specific gate and as a regulatory node that indirectly influences non-apoptotic cell death.

This distinction is important for the proposed model. Mitochondrial remodeling may directly alter apoptotic commitment while indirectly affecting other RCD modalities through changes in ROS production, metabolic flux, and lipid homeostasis. Conversely, compensatory antioxidant and metabolic responses may constrain these signals and preserve cellular viability. The resulting bidirectional coupling provides a mechanism through which mitochondrial state can influence the accessibility of multiple death programs without implying that all RCD pathways converge on mitochondria.

#### 3.2.4. Integration: From Coordinated Adaptations to Network Remodeling

Redox regulation, survival signaling, and mitochondrial state are therefore considered interconnected components of the proposed RCDN.

Directional signaling relationships documented in APL models support coupling among these modules: ROS → Akt (protective response), Akt → NRF2 → antioxidant (feedback loop), and mitochondria → ROS (NOX2-dependent loop) [11,13,21,29,48,52,59,62,64,67].

APL-specific molecular evidence supports the existence of these coupling relationships. Transglutaminase 2 (TG2) has been identified as a membrane-associated signaling hub that links PI3K, PTEN, and mTOR activity in APL cells. TG2 absence restores phospho-AKT and PI3K activity, thereby sensitizing APL cells to ATO-induced death [37]. This positions TG2 as a direct molecular bridge between survival signaling and death susceptibility. Similarly, NADPH oxidase 2 (NOX2) activation couples ATO-induced oxidative stress to mitochondrial superoxide formation in APL cells, providing a direct mechanistic link between the redox and mitochondrial modules [64]. PML-RARα itself modulates the PI3K/AKT pathway, with NLS-RARα overexpression promoting proliferation and inhibiting differentiation through PI3K/AKT signaling [11,12], linking the core oncoprotein directly to survival signaling reinforcement. The restoration of PML-NB organization by ATO [29,30,31,32] further connects nuclear stress sensing to cytoplasmic redox and mitochondrial regulation, establishing PML-NBs as a candidate upstream coordinator of RCDN coupling.

The coupling relationships described above are not static but evolve under sustained therapeutic pressure. In treatment-sensitive cells, ROS → Akt → NRF2 feedback remains transient and insufficient to override mitochondrial commitment [21,64]. Under persistent ATO/ATRA exposure, however, this feedback loop becomes reinforced through the upregulation of antioxidant enzymes [21,45,46] and sustained Akt phosphorylation [59,62], progressively raising the threshold for ROS-induced MOMP. The resulting high-threshold state is stabilized by reciprocal reinforcement: elevated ROS tolerance permits continued survival signaling, which in turn maintains mitochondrial integrity and antioxidant capacity [22]. With sustained ATO/ATRA exposure, transient adaptive responses may become more persistent through reinforcement of antioxidant capacity, Akt signaling, and mitochondrial protection.

This model provides a more precise interpretation of resistance heterogeneity. A resistant phenotype need not reflect the complete inactivation of a death pathway; instead, the relative weighting and coupling of upstream regulatory modules may shift the threshold for death engagement. Such remodeling could be reversible, partially reversible, or stabilized by feedback depending on cellular state and treatment history. The distinction between pathway activation and network state is therefore central: increased Akt activity, enhanced antioxidant capacity, or altered mitochondrial function may each represent components of resistance, but none alone demonstrates network remodeling.

**Figure 2:**
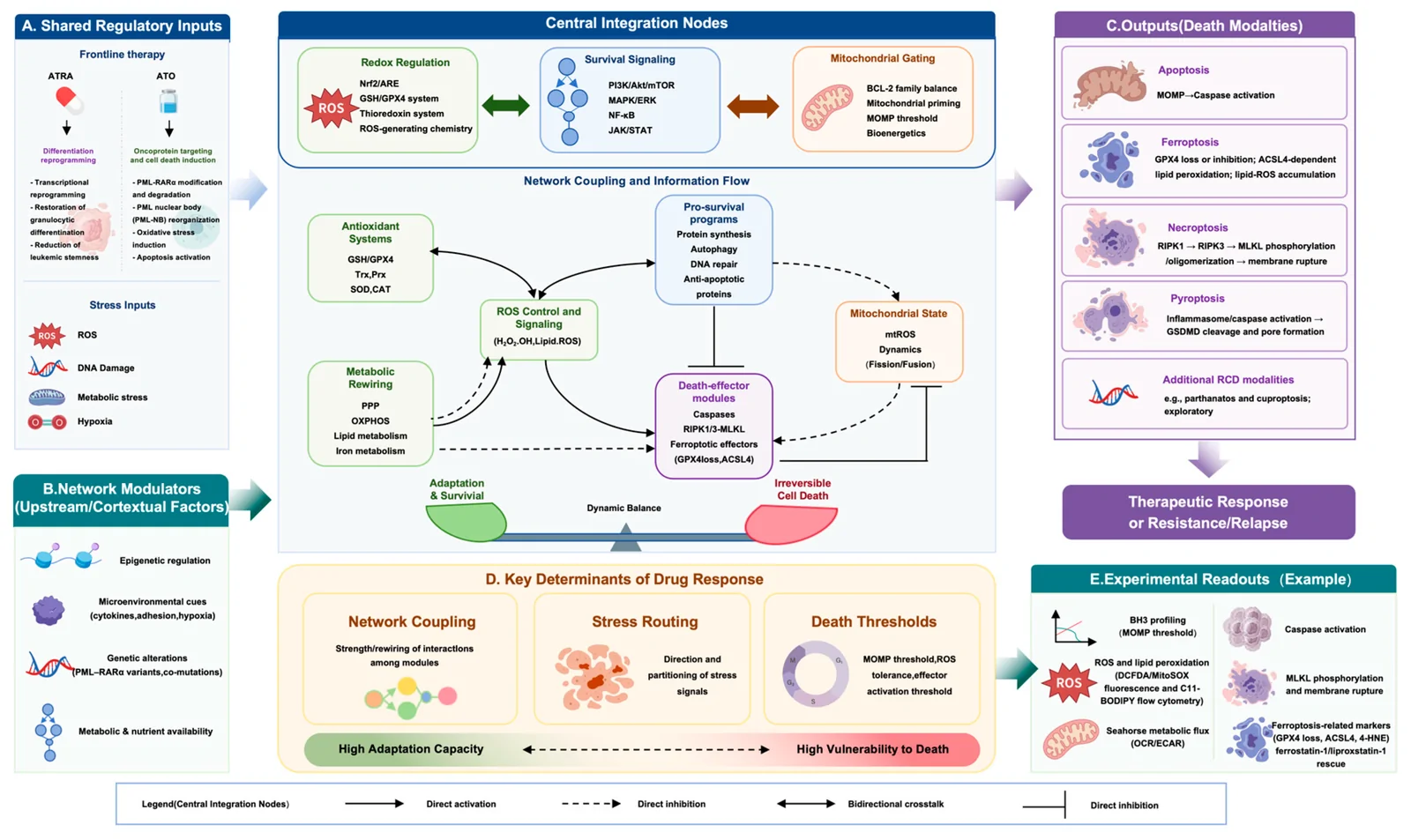
Proposed architecture of the RCDN in APL. The network integrates shared regulatory inputs, contextual modifiers, redox regulation, survival signaling, mitochondrial gating, and multiple death-effector modules. Solid and dashed arrows distinguish direct, indirect, and hypothesized relationships.

Accordingly, we interpret the available evidence as consistent with coordinated changes in redox regulation, survival signaling, mitochondrial function, and death execution, while recognizing that direct evidence for RCDN remodeling in APL remains limited. This interpretation is compatible with observations of metabolic adaptation during ATO resistance and with evidence that pharmacological manipulation of apoptotic dependence can modify responses to arsenic-based therapy [21,69,70,71,72]. The available evidence, however, does not establish RCDN remodeling as a mechanism of APL resistance. Instead, the framework identifies a set of experimentally testable predictions: resistant states would be expected to display coordinated changes across multiple death-related modules, perturbation of one module would be predicted to alter the response of others, and combined or sequential perturbations would be expected to produce non-additive responses that cannot be explained by individual pathway activity alone (Figure 2).

### 3.3. Context Dependence, Alternative Interpretations, and Candidate Upstream Modifiers

#### 3.3.1. Why the Same Molecular Change May Have Opposite Effects

A major limitation of a simple pathway narrative is that many resistance-associated signals are not intrinsically pro-survival or pro-death. ROS, PI3K/Akt signaling, autophagy, metabolic rewiring, and mitochondrial remodeling can exert opposing effects depending on cellular context. The framework therefore considers cellular state and response trajectories when interpreting these signals. The same molecular change may increase or decrease death susceptibility depending on redox buffering capacity, differentiation state, metabolic reserve, mitochondrial priming, treatment duration, and compensatory capacity [21,39,47].

Alternative explanations remain plausible. Apparent network remodeling may reflect independent adaptive responses occurring in parallel or may represent consequences rather than causes of resistance driven primarily by genetic or epigenetic alterations. Distinguishing primary RCDN remodeling from secondary adaptation therefore requires temporal and intervention-based evidence. Temporal and intervention-based experiments are needed to distinguish primary RCDN remodeling from secondary adaptation. Changes that precede stable resistance and whose early disruption prevents subsequent drug tolerance would support a causal role. In contrast, changes that consistently follow established resistance states would be more consistent with secondary adaptation.

Microenvironmental protection, immune interactions, and treatment-related factors may generate resistance phenotypes that are not fully captured by a cell-intrinsic model. The RCDN framework is primarily concerned with stress processing within leukemia cells and does not directly address differentiation syndrome or coagulopathy. Nevertheless, the bone marrow niche may act as an upstream modifier that shifts the intracellular network state without itself constituting an RCDN module.

Stromal cells, endothelial cells, adipocytes, and immune cells within the bone marrow niche may provide soluble, metabolic, and adhesion-dependent signals that increase stress tolerance [73]. C-X-C motif chemokine ligand 12 (CXCL12)–C-X-C motif chemokine receptor 4 (CXCR4) signaling and integrin-mediated adhesion may promote leukemic cell retention and activate survival pathways, including PI3K/Akt signaling [73]. Stromal interleukin-6 (IL-6) can activate signal transducer and activator of transcription 3 (STAT3) and enhance mitochondrial oxidative phosphorylation in AML cells, thereby increasing metabolic fitness and chemoresistance [74]. Within the RCDN framework, these niche-derived inputs could raise the death threshold by enhancing redox buffering, reducing mitochondrial priming, or sustaining survival signaling. Although these mechanisms have been described in hematological malignancies [73,74], their contribution to RCDN remodeling in APL remains to be established.

These considerations impose stringent criteria for validating the RCDN model. RCDN remodeling is best inferred when functional interactions among modules are demonstrated, when perturbation of one node alters the functional effect of another, and when integrated network-state measurements provide greater explanatory or predictive power than individual pathway markers. Without such evidence, the RCDN framework is best regarded as an organizing construct rather than a distinct mechanistic model (Table 3) [22,75].

#### 3.3.2. PML-NBs as Candidate Upstream Modifiers

PML nuclear bodies (PML-NBs) are dynamic, membraneless nuclear structures involved in DNA damage responses, senescence, apoptosis, and cellular stress adaptation [76,77,78]. In APL, PML–RARα disrupts normal PML-NB organization, whereas ATO-mediated degradation of PML–RARα contributes to the restoration of PML-NB architecture during therapeutic response [3,4,59,60,79]. These observations establish the relevance of PML-NBs to APL biology but do not demonstrate that PML-NBs function as upstream integrators of the RCDN.

A more cautious interpretation is that PML-NBs may modify the coupling between nuclear stress-sensing and downstream death-regulatory pathways. PML-associated processes have been linked to p53 regulation, stress responses, DNA damage signaling, and mitochondrial apoptotic control [76,77,78]. PML-NB dynamics may therefore influence redox handling, survival signaling, and mitochondrial priming.

Several lines of evidence support a role for PML-NBs in coupling nuclear stress-sensing to downstream redox, survival, and mitochondrial regulation. First, PML functions as a direct ROS sensor: PML−/− cells accumulate ROS, whereas PML expression decreases ROS levels, and PML-NB biogenesis couples ROS sensing to p53-dependent transcriptional responses [31]. PML exerts basal antioxidant properties while also driving oxidative stress-induced changes in cell survival and metabolism [31]. Second, PML negatively regulates the PI3K/Akt/mTOR pathway through multiple mechanisms: PML recruits PP2A phosphatases to PML-NBs, promoting PP2A-dependent AKT inhibition [59]; PML activates PTEN, further suppressing PI3K signaling [59]; and PML directly inhibits mTOR activation [77]. Third, PML-NBs modulate mitochondrial apoptotic priming through both nuclear and cytoplasmic routes: PML stabilizes p53 within PML-NBs, enhancing p53-dependent pro-apoptotic transcription [76]; cytoplasmic PML can translocate to mitochondria-associated membranes (MAMs), regulating Ca^2+^ transfer to mitochondria and subsequent loss of mitochondrial membrane potential [77]; and PML overexpression increases the frequency of apoptotic cells, nuclear condensation, and the loss of mitochondrial membrane potential, accompanied by the regulation of Bcl-2 family proteins and ROS levels [31,42,50].

However, the proposed PML–FOXO3a/NRF2 axis and PML–BCL-2 family interactions have not been established as a unified regulatory circuit in APL resistance. These relationships are best regarded as mechanistic possibilities rather than established features of resistant APL.

PML-NBs are therefore best treated as a candidate upstream modifier rather than a proven core component of the RCDN. The relevant tests are direct: following ATO exposure, do the extent and kinetics of PML-NB restoration correlate with independently measured death susceptibility at the single-cell level? Does genetic or pharmacological manipulation of PML-NB assembly alter redox processing, Akt feedback, or mitochondrial death thresholds? If these relationships cannot be demonstrated, PML-NBs may still contribute to APL treatment response, but their proposed role as an RCDN integrator remains unsupported [59,60,80,81].

### 3.4. Experimental Validation Strategy

#### 3.4.1. Functional Assays of Death Susceptibility

The primary readout for testing RCDN remodeling is functional death susceptibility rather than viability alone. Although viability-based half-maximal inhibitory concentration (IC50) values remain useful, they integrate multiple processes, including drug exposure, proliferation, and stress adaptation. MOMP, cytochrome c release, and caspase activation provide execution-associated readouts of apoptosis [27,65]. In contrast, lipid peroxidation and mixed lineage kinase domain-like protein (MLKL) phosphorylation indicate ferroptotic and necroptotic pathway engagement, respectively, and require orthogonal confirmation through membrane-integrity assays and pathway-specific rescue [26,28,33,38]. These measurements can be complemented by the assessment of ROS dynamics, antioxidant capacity, Akt activity, mitochondrial priming, and relevant death-effector states [39,59,75].

A cell death sensitivity profile could be generated using a focused panel of mechanistically distinct perturbations rather than by testing every recognized form of regulated cell death [26]. The initial panel should prioritize apoptosis because ATO-induced oxidative stress, mitochondrial dysfunction, and caspase activation provide the strongest direct evidence in APL [13,29,30,31,32,37,64]. Apoptotic sensitivity could be assessed using BH3 profiling, MOMP, cytochrome c release, caspase activation, and rescue with caspase inhibitors or interventions targeting BCL-2 family proteins [27,61,63,72]. Ferroptotic sensitivity could be examined by combining measurements of lipid peroxidation with rescue by ferrostatin-1 or liproxstatin-1 and the assessment of GPX4 dependency [25,28,38]. Regulated necrotic responses should be examined when preliminary findings suggest pathway engagement and confirmed using pathway-specific markers together with pharmacological or genetic rescue [26,33,59]. Pyroptosis and other less directly implicated modalities need not be routinely included unless preliminary evidence indicates their relevance [24,26].

Available evidence in APL most clearly supports ATRA-induced differentiation and ATO-associated apoptosis, with the combined regimen promoting PML–RARα degradation and leukemic cell clearance through complementary mechanisms [3,4,12,13,29,30,31,32,37]. Evidence that ATRA/ATO directly induces ferroptosis, necroptosis, pyroptosis, or other non-apoptotic death programs in APL remains limited. These modalities are therefore considered candidate escape routes or collateral vulnerabilities to be tested, rather than established effects of standard ATRA/ATO therapy [23,24,25,26,28,33,38].

Single-cell approaches are particularly valuable because population-level measurements can obscure resistant subpopulations, intermediate stressed-but-viable states, and heterogeneous responses to similar stress inputs. Combining redox sensors, phospho-signaling measurements, mitochondrial reporters, BH3 profiling, and modality-specific death readouts could therefore characterize RCDN remodeling as a dynamic state rather than a static marker profile and link heterogeneity in death thresholds to the underlying cellular state [27,75,78].

#### 3.4.2. Tests That Distinguish RCDN from Simpler Models

The critical objective is not to demonstrate the activation of individual RCD pathways, but to determine whether their functional interactions provide explanatory power beyond pathway-specific models. Several experimental comparisons can distinguish RCDN remodeling from simpler pathway-specific models [26,33,38]. First, sensitive and resistant APL models can be tested using mechanistically distinct death stimuli. Coordinated but non-identical changes in susceptibility across apoptosis, ferroptosis, and regulated necrotic pathways would support a network-level alteration, whereas changes restricted to a single modality would favor a pathway-specific explanation. Second, calibrated ROS inputs can be applied to compare downstream responses at matched intracellular ROS levels. Divergent adaptive responses or delayed death execution despite comparable ROS exposure would support the altered processing of oxidative signals rather than differences in ROS generation alone [39,45,46,47,54].

Third, pulse–washout inhibition of PI3K/Akt or other feedback nodes could test whether the resistant state exhibits functional memory. Rapid recovery of the original phenotype after removal of the perturbation would favor a reversible pathway effect, whereas persistent changes in death susceptibility would be more consistent with feedback stabilization [21,22,59,62,63]. Fourth, combined perturbations can be evaluated for non-additive effects. Predominantly additive responses would provide limited evidence for network coupling, whereas synergistic or sequence-dependent responses would be more consistent with coupled state regulation [21,22,59,62,63].

Finally, as discussed in Section 3.4.1, temporal perturbation experiments can help determine whether RCDN remodeling precedes the establishment of stable resistance. Disrupting candidate RCDN nodes at early versus late stages of ATRA/ATO exposure could help distinguish a primary causal contribution from secondary adaptation. Longitudinal measurements combining molecular state, functional death thresholds, and drug response would provide a stronger test of this distinction than endpoint pathway measurements alone [21,22,78].

**Figure 3:**
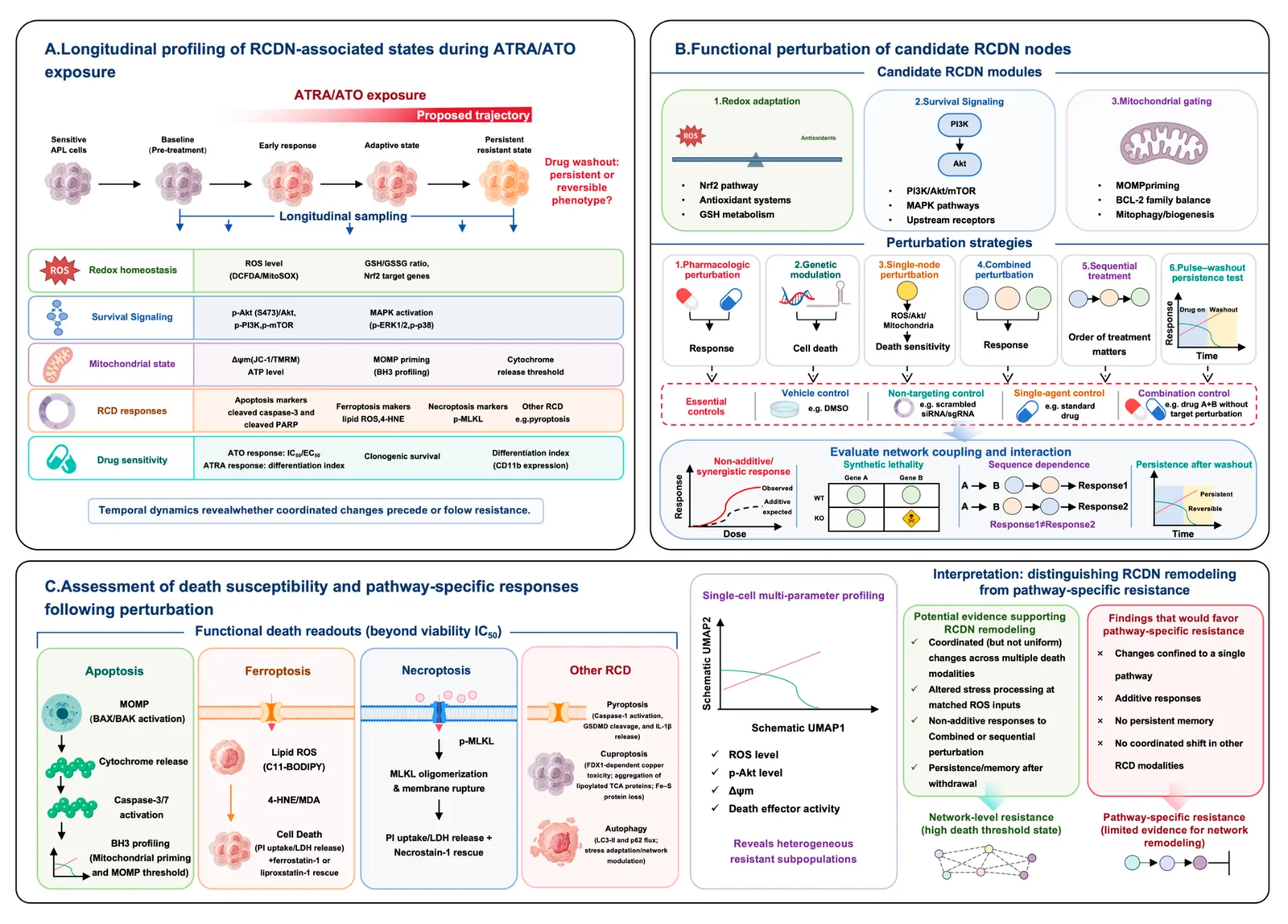
Proposed experimental strategies for testing RCDN remodeling in APL resistance. The strategy combines longitudinal profiling, functional perturbation, modality-specific death assays, single-cell measurements, and persistence testing after drug washout.

Where feasible, these analyses can be extended to patient-derived models. Such studies could provide exploratory validation through BH3 profiling, redox-response assays, and targeted perturbation, while recognizing practical limitations related to sample availability, cellular heterogeneity, assay standardization, and cost. Patient-derived studies are therefore best considered mechanistic validation rather than clinical decision tools (Figure 3) [27,75,76,78].

### 3.5. Tan IIA as an Experimental Probe of RCDN Remodeling

Tan IIA, a natural diterpenoid isolated from Salvia miltiorrhiza, serves as a valuable experimental tool for investigating mechanisms of cell death susceptibility and resistance. Its well-characterized molecular targets and multifaceted effects provide a suitable context for exploring the RCDN remodeling framework.

Evidence supporting its relevance is stratified into three levels: (1) direct evidence from APL studies [82], highlighting Tan IIA’s effects on redox balance, mitochondrial regulation, and apoptosis induction; (2) broader evidence from acute myeloid leukemia (AML), demonstrating similar effects in various AML subtypes [83]; and (3) non-APL contexts, including its modulation of survival signaling pathways, ROS processing, and cell death thresholds in diverse cancer models [84,85,86,87,88]. Collectively, these studies illustrate Tan IIA’s capacity to probe multi-dimensional network changes central to RCDN remodeling.

However, the translational value of Tan IIA is constrained by poor aqueous solubility, low oral bioavailability, rapid systemic clearance, and uncertain exposure within the bone marrow compartment [87,88,89]. Future development could proceed along two complementary routes. First, semi-synthetic analogs or prodrug derivatives could be optimized to improve solubility, metabolic stability, and target engagement while retaining the multi-node perturbational properties of the parent compound. Second, formulation-based approaches—including lipid nanoparticles, liposomes, polymeric carriers, and ligand-directed nanocarriers—could increase systemic exposure and potentially enhance delivery to leukemic cells or the marrow niche. Indeed, biotinylated lipid-coated mesoporous silica nanoparticles have been reported to improve the bioavailability and anti-leukemia activity of Tan IIA, providing proof of concept for formulation-based optimization [87]. Nevertheless, these approaches will require pharmacokinetic–pharmacodynamic characterization, marrow-distribution studies, toxicity assessment, and validation in resistant APL models before clinical relevance can be inferred. Tan IIA should therefore remain positioned primarily as a preclinical network perturbation probe, while optimized analogs and delivery systems may provide future routes toward therapeutic development.

**Figure 4:**
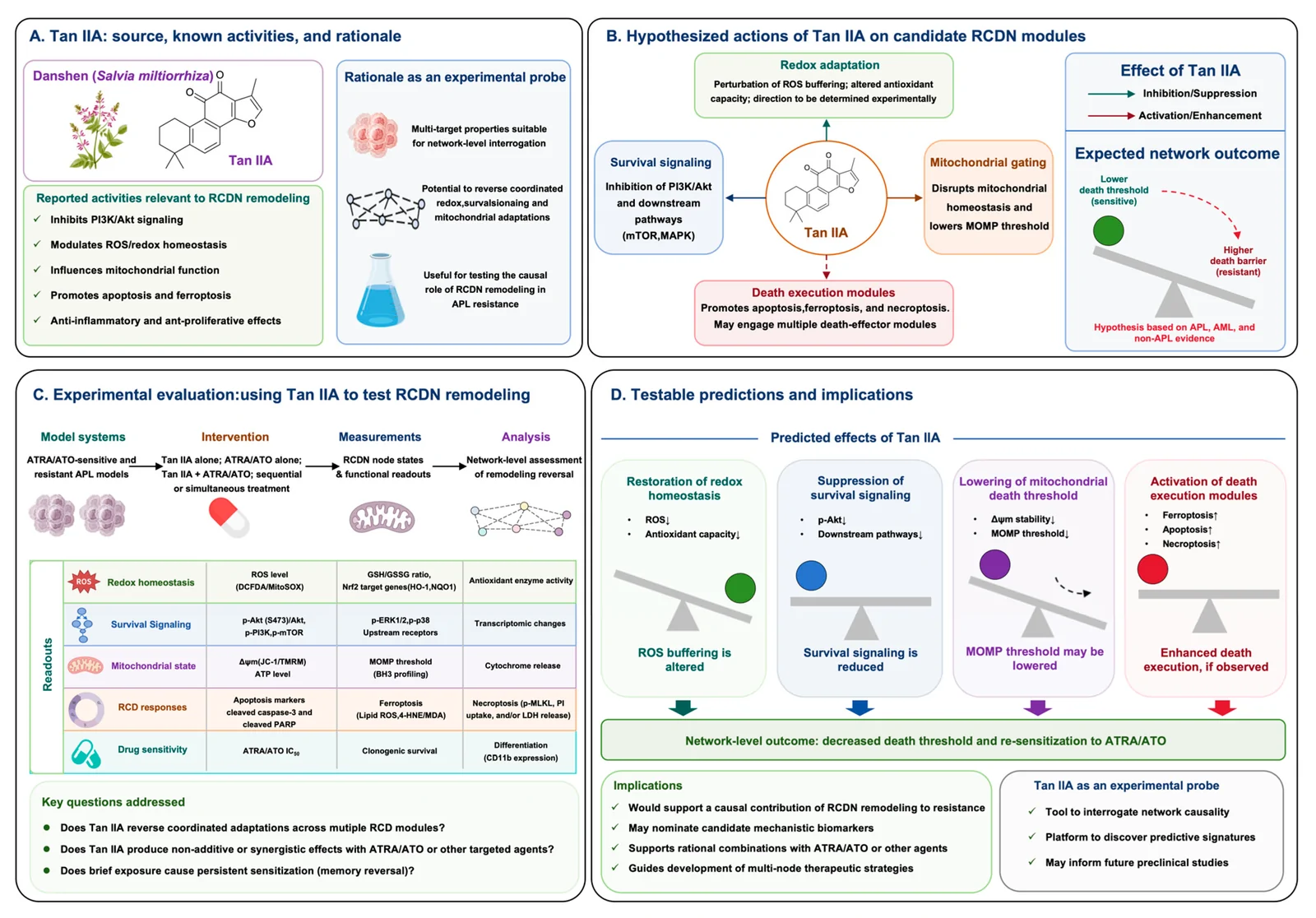
Tan IIA as a preclinical experimental probe of candidate RCDN remodeling. Reported activities are distinguished from hypothesized network effects and proposed experimental readouts. The predicted outcomes are testable hypotheses rather than established therapeutic effects.

These properties support the use of Tan IIA as a preclinical probe for examining functional interactions among redox adaptation, survival signaling, and mitochondrial regulation. Comparative testing of Tan IIA, optimized analogs, and targeted formulations could further determine whether improved exposure preserves or enhances its capacity to perturb multiple RCDN nodes (Figure 4 and Table 4).

## 4. Discussion

Redox regulation, Akt signaling, mitochondrial function, and cell death execution each contribute to APL treatment response, but their interactions during resistance remain unclear. We suggest that the connections among these processes, rather than a defect in a single pathway, may contribute to resistant states that cannot be fully explained by pathway-specific mechanisms. Future experiments should test whether these coordinated changes represent RCDN remodeling or a broader form of cellular adaptation.

## 5. Conclusions

The RCDN framework considers how redox adaptation, survival signaling, mitochondrial gating, and death-effector pathways interact to shape death thresholds in acute promyelocytic leukemia. It extends beyond single-pathway explanations by proposing that resistance may reflect changes in susceptibility to several death programs and differences in how cells process comparable therapeutic stress. Testing this possibility will require longitudinal profiling and functional perturbation in patient-derived models that reproduce relevant features of the bone marrow microenvironment. Such studies may determine whether RCDN states emerge before resistance and contribute to its maintenance, and whether RCDN measurements provide information about treatment response beyond that offered by established genetic markers.

## Figures and Tables

**Table 1:** Conceptual boundaries of the RCDN framework: comparison with related frameworks.

Concept	Core Focus	Level of Analysis	Relationship to the RCDN Framework
RCD crosstalk	Molecular and functional interactions between RCD modalities	Interactions between death pathways	RCD crosstalk describes interactions between individual death pathways, whereas the RCDN framework also considers shared regulatory inputs, stress routing, and changes in death thresholds.
Mitochondrial priming	Proximity to MOMP and commitment to intrinsic apoptosis, commonly assessed by BH3 profiling	Mitochondrial, apoptosis-specific functional readout	Mitochondrial priming represents the apoptosis-specific component of the broader RCDN death threshold; it does not directly measure susceptibility to non-apoptotic death programs.
Drug-tolerant persister state	Reversible survival state adopted by a subpopulation of cells during treatment	Cellular phenotype	The drug-tolerant persister state describes a surviving cellular phenotype, whereas the RCDN framework proposes how coupled regulatory changes may generate or maintain such a state.
Non-genetic/adaptive resistance	Resistance arising without stable genetic alterations	Broad mechanistic category	RCDN remodeling is proposed as one possible network-level mechanism within the broader category of non-genetic or adaptive resistance.
PANoptosis	Coordinated engagement of pyroptotic, apoptotic, and necroptotic machinery within an integrated cell death process	Death-execution machinery	PANoptosis concerns the engagement of specific death-execution pathways, whereas the RCDN framework examines how regulatory and execution modules collectively shape stress responses and death susceptibility.
RCD signaling network maps	Static representation of RCD-related molecules, pathway and interactions	Network topology	Static maps identify network components and possible connections, whereas the RCDN framework focuses on functional network states, coupling, and responses to defined perturbations.

**Table 2:** Component-specific evidence for the proposed RCDN remodeling framework in APL.

RCDN Component/Hypothesis	Direct APL Evidence	AML Evidence	Evidence from Other Cancer Models	Evidence Level	Role in the Present Framework
PML-RARα degradation and ATO response	PML-RARα degradation is required for ATRA/ATO activity; PML-RARα mutations have been associated with ATO resistance [2,3,4,13,14,29,30]	—	—	High	Established APL mechanism; provides the molecular starting point but does not fully explain resistance
PML-NB restoration	ATO-mediated PML-RARα degradation is associated with restoration of PML-NB organization [4,29,30,31,32]	—	—	High for APL biology; low for RCDN integration	Candidate upstream modifier of the RCDN rather than a proven network integrator
Redox adaptation/ROS signaling	Oxidative stress and ROS-related responses have been implicated in APL treatment response and resistance [13,21,30,31,36]	ROS-mediated mitochondrial damage has been linked to chemoresistance in AML [47]	ROS can activate survival or death responses depending on cellular context [39,45,46,48,49,50]	Moderate–High	Candidate regulatory module controlling stress routing and death thresholds
PI3K/Akt survival signaling	PI3K/Akt-related signaling has been implicated in APL resistance and treatment response [11,12,21]	PI3K/Akt signaling contributes to survival and metabolic adaptation in AML [47,50,56,57]	Context-dependent effects of Akt signaling have been described across cancers [54,55,56,57]	Moderate	Candidate feedback node connecting stress adaptation with death susceptibility
Mitochondrial regulation	Mitochondrial dysfunction and apoptotic regulation have been implicated in APL treatment response [13,21,27,30,31,36]	Mitochondrial metabolism and oxidative phosphorylation (OXPHOS) are linked to leukemia persistence and therapeutic resistance [21,47]	Mitochondrial priming and BCL-2 family regulation determine apoptotic sensitivity across cancers [21,27,36,41,54,55]	Moderate–High	Proposed functional gate regulating apoptotic commitment and cross-pathway coupling
Autophagy as a stress-adaptation process	Autophagy has been directly implicated in APL treatment response and resistance	Autophagy contributes to survival adaptation in leukemia models [44]	Tan IIA-induced autophagy has been reported in non-APL cancer models [23,44,51]	Moderate	Network-modulating process rather than a canonical RCD modality in the present model
Ferroptosis/lipid peroxidation	Direct APL evidence remains limited	SLC7A11/GPX4-related ferroptosis regulation has been reported in AML [25,28,38,50]	Ferroptosis mechanisms are extensively established in other cancers [25,28,38,42,48,52]	Moderate for AML; limited for APL	Candidate alternative death route and cross-pathway output
Non-apoptotic RCD crosstalk	Direct integrated evidence in APL remains limited	Individual death pathways have been investigated in AML [16,21,22]	Crosstalk among apoptosis, ferroptosis, necroptosis, and pyroptosis is established across cancer models [24,25,26,33,35,38]	Limited–Moderate	Provides biological rationale for the network concept but remains a hypothesis in APL
RCDN remodeling itself	No direct experimental validation currently established	Indirect support from heterogeneous resistance and metabolic adaptation studies [18,21,22]	Network plasticity and non-genetic resistance provide conceptual support [17,18,19,20,34,53]	Hypothesis	Central conceptual proposition requiring experimental validation
Tan IIA as a multi-node perturbation	Tan IIA induces apoptosis and modulates autophagy in APL models	Tan IIA regulates AML proliferation and apoptosis	ROS-, Akt-, MAPK-, and autophagy-related effects have been reported in other cancer models [23,44,47,50,51]	Moderate; strongest for APL apoptosis	Experimental probe for testing multi-node RCDN perturbation, not yet a validated RCDN-directed therapy

**Table 3:** Evidence tiers and their relevance to RCDN remodeling in APL.

Evidence Level	Evidence Source	What It Supports	Interpretation in This Perspective
Level 1	APL-specific studies (cell lines, patient samples, clinical data) [1,2,3,4,5,6,7,8,11,12,13,14,21,29,30,31,32,69,72]	ATO-induced oxidative stress and PML-RARα degradation; ATRA-induced differentiation and ATO-associated apoptosis; PML-NB biology and p53 activation; ATRA/ATO treatment response and clinical outcomes; PML-RARα mutations and resistance	Direct evidence: APL-specific experimental or clinical observations that anchor the framework in disease-relevant biology
Level 2	Broader AML studies [16,17,18,19,20,47,50,51,56,57,59,61,62,64,70,71]	PI3K/Akt signaling and survival adaptation; mitochondrial metabolism and OXPHOS; metabolic remodeling and non-genetic drug tolerance; ferroptosis regulation (SLC7A11/GPX4); drug-tolerant persister states	Supportive evidence: Mechanistically relevant but not APL-specific; provides rationale for testing analogous processes in APL
Level 3	Other cancer systems [17,18,19,20,22,23,24,25,26,27,28,33,34,35,38,39,40,41,42,43,44,45,46,48,49,50,51,52,53,54,55,56,57,58,59,60,63,65,66,67,68,72,75]	ROS signaling and redox adaptation; RCD plasticity and death thresholds; ferroptosis–apoptosis crosstalk; network-level stress responses; mitochondrial regulation and calcium signaling; context-dependent Akt and MAPK effects	Contextual evidence: Establishes mechanistic plausibility and generates hypotheses for APL-specific investigation
Level 4	RCDN framework proposed here [16,17,18,19,20,22,23,24,25,26,27,28,33,34,35,36,38,39,44,53,61,62,72,75]	Network coupling among redox, survival, mitochondrial, and death-effector modules; stress routing and death-threshold modulation; coordinated remodeling across regulatory modules; distinction from RCD plasticity and simple pathway switching	Conceptual proposition: Testable hypothesis requiring direct experimental validation in APL models; the proposed approach to cell death sensitivity profiling is described in Section 3.4.1

**Table 4:** Therapeutic strategies targeting RCDN remodeling in APL.

Therapeutic Strategy	Representative Targets/Interventions	Mechanistic Rationale for RCDN Remodeling	Evidence Level in APL	Translational Implications	Representative References
Restoration of mitochondrial apoptotic sensitivity	BCL-2 inhibition (venetoclax), MCL-1 inhibition, BH3 mimetics	Reduces anti-apoptotic buffering capacity, lowers mitochondrial threshold for MOMP, restores apoptotic priming in resistant cells	Emerging evidence (APL-specific studies available; stronger evidence from AML models)	May enhance the elimination of residual or ATO-resistant leukemia cells by restoring mitochondrial apoptosis	[69,70,71,72]
ROS-dependent death amplification	Arsenic trioxide (ATO), redox modulators, glutathione metabolism targeting	Increases intracellular ROS beyond adaptive survival capacity, induces PML–RARα degradation, mitochondrial dysfunction, and apoptotic commitment	Strong evidence	Clinically validated mechanism exploiting APL-specific oxidative vulnerability	[3,4,13,29,30]
Targeting antioxidant adaptation	NRF2 pathway inhibition, glutathione depletion, thioredoxin system targeting	Disrupts antioxidant buffering systems that allow leukemia cells to tolerate therapy-induced oxidative stress	Preclinical evidence	May overcome redox adaptation and increase sensitivity to ATO-based therapy	[21,45,46]
Ferroptosis induction and metabolic reprogramming	GPX4 inhibition, SLC7A11/system Xc^−^ inhibition, iron metabolism modulation	Converts metabolic adaptation into iron-dependent lipid peroxidation and ferroptotic vulnerability	Limited direct evidence in APL; exploratory evidence from AML models	Potential strategy for targeting resistant populations with altered redox metabolism	[25,28,38]
Autophagy modulation	mTOR inhibition, chloroquine/hydroxychloroquine, ATG pathway targeting	Regulates stress adaptation pathways that maintain leukemia survival during therapeutic pressure	Moderate evidence	May enhance differentiation therapy response; effects depend on whether autophagy functions as a survival or death mechanism	[44,87,88]
Mitochondrial metabolic vulnerability targeting	OXPHOS inhibitors, mitochondrial metabolism regulators	Disrupts metabolic plasticity and mitochondrial adaptation supporting leukemia persistence	Emerging evidence	Provides strategy to eliminate metabolically adapted resistant clones	[61,62,64]
Inflammatory regulated cell death modulation	NLRP3 inflammasome regulation, caspase-1/GSDMD pathway modulation	Alters inflammatory cell death signaling and leukemia–microenvironment interactions	Limited evidence in APL	Potential future approach for regulating immune-mediated leukemia clearance	[26,35,38]
Epigenetic reprogramming of death susceptibility	HDAC inhibitors, DNA methylation inhibitors, transcriptional regulators	Restores transcriptional programs controlling differentiation, apoptosis, and cellular stress responses	Moderate evidence in AML; limited APL-specific application	May reverse resistant cellular states and enhance differentiation-based therapy	[10,79]
Enhancement of immunogenic cell death and immune clearance	ICD inducers, immune activation strategies, checkpoint modulation	Promotes DAMP release, antigen presentation, and immune-mediated elimination of residual leukemia cells	Emerging evidence	May provide future strategies for minimal residual disease control	[26,35]
Multi-layer network modulation	Tan IIA (illustrative probe), other multi-target compounds	Simultaneously perturbs ROS, PI3K/Akt signaling, mitochondrial function, and PML-NB/SUMOylation pathways	Hypothetical (preclinical evidence in non-APL models)	Proof of concept for testing network-level threshold lowering; not a validated therapeutic candidate	[82,83,84,85,86,87,88,89,90]

## Data Availability

No new data were created or analyzed in this study. Data sharing is not applicable to this article.

## Abbreviations

The following abbreviations are used in this manuscript:

Akt	protein kinase B
AML	acute myeloid leukemia
APL	acute promyelocytic leukemia
ATO	arsenic trioxide
ATRA	all-trans retinoic acid
BAK	BCL-2 homologous antagonist/killer
BAX	BCL-2-associated X protein
BCL-2	B-cell lymphoma 2
BH3	BCL-2 homology 3
CXCL12	C-X-C motif chemokine ligand 12
CXCR4	C-X-C motif chemokine receptor 4
DAMPs	damage-associated molecular patterns
DTP	drug-tolerant persister
GPX4	glutathione peroxidase 4
GSH	glutathione
HDAC	histone deacetylase
IC50	half-maximal inhibitory concentration
ICD	immunogenic cell death
IL-6	interleukin-6
LC3	microtubule-associated protein 1 light chain 3
LDH	lactate dehydrogenase
MAMs	mitochondria-associated membranes
MAPK	mitogen-activated protein kinase
MLKL	mixed lineage kinase domain-like protein
MOMP	mitochondrial outer membrane permeabilization
mTOR	mechanistic target of rapamycin
NF-κB	nuclear factor kappa B
NOX2	NADPH oxidase 2
NRF2	nuclear factor erythroid 2-related factor 2
OXPHOS	oxidative phosphorylation
PI3K	phosphoinositide 3-kinase
PML	promyelocytic leukemia protein
PML-NBs	promyelocytic leukemia nuclear bodies
PML–RARα	promyelocytic leukemia–retinoic acid receptor alpha fusion protein
PP2A	protein phosphatase 2A
PTEN	phosphatase and tensin homolog
RARα	retinoic acid receptor alpha
RCD	regulated cell death
RCDN	regulated cell death network
ROS	reactive oxygen species
SLC7A11	solute carrier family 7 member 11
STAT3	signal transducer and activator of transcription 3
SUMO	small ubiquitin-like modifier
Tan IIA	Tanshinone IIA
TG2	transglutaminase 2
